# Comprehensive Analysis of a Zinc Finger Protein Gene–Based Signature with Regard to Prognosis and Tumor Immune Microenvironment in Osteosarcoma

**DOI:** 10.3389/fgene.2022.835014

**Published:** 2022-02-25

**Authors:** Xiangran Sun, Di Zheng, Weichun Guo

**Affiliations:** Department of Orthopedics, Renmin Hospital of Wuhan University, Wuhan, China

**Keywords:** zinc finger protein genes, signature, prognosis, osteosarcoma, nomogram

## Abstract

Osteosarcoma is the most common malignant bone tumor that seriously threatens the lives of teenagers and children. Zinc finger (ZNF) protein genes encode the largest transcription factor family in the human genome. Aberrant expressions of ZNF protein genes widely occur in osteosarcoma, and these genes are therefore attractive biomarker candidates for prognosis prediction. In this study, we conducted a comprehensive analysis of ZNF protein genes in osteosarcoma and identified prognosis-related ZNF protein genes. Then, we constructed a prognostic signature based on seven prognosis-related ZNF protein genes and stratified patients into high- and low-risk groups. The seven genes included *MKRN3*, *ZNF71*, *ZNF438*, *ZNF597*, *ATMIN*, *ZNF692*, and *ZNF525*. After validation of the prognostic signature in internal and external cohorts, we constructed a nomogram including clinical features such as sex and age and the relative risk score based on the risk signature. Functional enrichment analysis of the risk-related differentially expressed genes revealed that the prognostic signature was closely associated with immune-related biological processes and signaling pathways. Moreover, we found significant differences between the high- and low-risk groups for the scores of diverse immune cell subpopulations, including CD8^+^ T cells, neutrophils, Th1 cells, and TILs. Regarding immune function, APC co-inhibition, HLA, inflammation promotion, para-inflammation, T-cell co-inhibition, and the type I IFN response were significantly different between the high- and low-risk groups. Of the seven ZNF protein genes, lower expressions of *ATMIN*, *MKRN3*, *ZNF71*, *ZNF438*, and *ZNF597* were correlated with a high risk, while higher expressions of *ZNF525* and *ZNF692* were associated with a high risk. The Kaplan–Meier survival analysis suggested that lower expressions of *ATMIN*, *ZNF438*, and *ZNF597* and the higher expression of *ZNF692* were associated with worse overall survival in osteosarcoma. In conclusion, our ZNF protein gene–based signature was a novel and clinically useful prognostic biomarker for osteosarcoma patients.

## Introduction

Osteosarcoma is the most common malignant bone tumor that seriously threatens the lives of teenagers and children, with a second incidence peak in the elderly over the age of 60 years (Ritter and Bielack, 2010). In the last few decades, huge progresses in the diagnosis and management of osteosarcoma have been made (Kager et al., 2017; Chen et al., 2020). Surgical resection combined with neo-adjuvant chemotherapy is currently the standard treatment for patients with osteosarcoma, and it increases the 5-year overall survival from 20 to 70% for patients with localized disease (Kansara et al., 2014; Harrison et al., 2018). However, the 5-year overall survival is still less than 20% for patients with metastatic or recurrent osteosarcoma and has remained almost unchanged over the past 30 years (Kansara et al., 2014). Additionally, resistance to chemotherapy regimens often occurs due to the high heterogeneity in the genome of osteosarcoma, and it seriously impacts the prognosis of patients with osteosarcoma (Gianferante et al., 2017; Sayles et al., 2019). Despite the existence of some prognostic biomarkers in osteosarcoma, it is still unachievable to accurately predict the prognosis of patients with osteosarcoma. Therefore, the development of novel risk stratification methods, as well as identification of novel prognostic biomarkers, are clinically useful.

The zinc finger (ZNF) protein genes encode the largest family of regulatory proteins with zinc ion–binding finger-like domains in the human genome (Laity et al., 2001). The functions of these ZNF proteins are extremely diverse, and they can interact with RNA, DNA, or proteins to participate in various cellular processes including transcriptional regulation, mRNA stability, and protein degradation (Leon and Roth, 2000; Fu and Blackshear, 2017; Wang and Zheng, 2021). Over the last decades, increasing number of researches have revealed the vital role of ZNF proteins in oncogenesis and its progression (Jen and Wang, 2016; Cassandri et al., 2017; Ye et al., 2019). Aberrant expressions of ZNF proteins or genes have been found in malignant tumors and might serve as diagnostic or prognostic biomarkers. For example, Poirier et al. (2021) reported that *ZNF768* was elevated in lung adenocarcinoma when compared to normal lung tissue and was associated with clinicopathological features. *In vitro* experiments suggested that depletion of *ZNF768* severely impairs proliferation in several lung cancer cell lines. A further example includes *ZNF471*, which acts as a tumor suppressor in cervical cancer. Hypermethylation of the *ZNF471* gene promoter and low *ZNF471* expression was correlated with poor overall and recurrence-free survival in cervical cancer (Bhat et al., 2021).

In this study, we conducted a comprehensive analysis of ZNF protein genes in osteosarcoma. Prognosis-related ZNF protein genes were identified and further used to construct a prognostic signature in osteosarcoma. We found that our novel ZNF protein gene–based signature could act as a risk stratification method and a prognostic biomarker. Meanwhile, we also developed a prognostic nomogram comprising clinical features such as gender and age and the relative risk score based on the risk signature. Additionally, we found that our ZNF protein gene–based signature was closely associated with immune cell infiltration and immune function. Taken together, our ZNF protein gene–based signature was a novel and clinically useful prognostic biomarker for osteosarcoma patients, and our findings shed light on the potential role of ZNF proteins in the pathogenesis of osteosarcoma.

## Materials and Methods

### Data Collection and Zinc Finger Protein Genes

The RNA-seq data and corresponding clinical information of patients with osteosarcoma were downloaded from The Cancer Genome Atlas (TCGA) database (https://portal.gdc.cancer.gov/). The GSE21257 data set, containing the gene expression matrix data from a total of 55 osteosarcoma samples and clinical data, was collected from the Gene Expression Omnibus (GEO) database (https://www.ncbi.nlm.nih.gov/geo/) for external validation. Patients without follow-up time or incomplete clinical data were excluded in the subsequent analysis. The list of ZNF protein genes was obtained from the UniProt database (https://www.uniprot.org/), and the ZNF protein genes were adopted to a unified terminology in the gene expression matrixes from the TCGA and the GEO databases.

### Identification of Prognosis-Related Zinc Finger Protein Genes in Osteosarcoma

Prognosis-related ZNF protein genes were identified by performing univariate Cox regression analysis. Genes with a *p*-value <0.05 in the univariate Cox regression analysis were regarded as prognosis-related ZNF protein genes.

### Construction and Validation of a Zinc Finger Protein Gene–Based Signature

The TCGA osteosarcoma cohort, hereafter referred to as the entire cohort, was randomly separated into a training cohort and a testing cohort at a ratio of approximately 1:1. Then, in the training cohort, the prognosis-related ZNF protein genes were subjected to the least absolute shrinkage and selection operator (LASSO) regression analysis to eliminate collinearity among these genes. Subsequently, the multivariate Cox regression analysis was conducted using the survival package in R to calculate the regression coefficients, and we ultimately constructed a ZNF protein gene–based signature in training cohort. The risk scores of the patients in the testing cohort, entire cohort, and GSE21257 cohort were calculated based on the signature and the formula as follows:

$$\text{risk score }=\overset{n}{\underset{i=1}{\sum }}\left(Coefi*Expi\right)$$

The Coefi is the regression coefficients of the selected genes, and the Expi is the expression levels of the selected genes. Using the median risk score value in the training cohort, patients in the testing cohort, entire cohort, and GSE21257 cohort were classified into high- and low-risk groups. The clinicopathological characteristics of the osteosarcoma patients in the TCGA entire cohort and the GSE21257 cohort are shown in Table 1. The Kaplan–Meier survival analysis was performed using the *survival* and *survminer* packages in R to compare the overall survival of patients in the high- and low-risk groups. Time-dependent receiver operating characteristic (ROC) curve analysis was conducted using the *survivalROC* package in R to verify the accuracy of the ZNF protein gene–based signature in predicting the prognosis of patients with osteosarcoma.

**TABLE 1:** Clinicopathological characteristics of osteosarcoma patients in high- and low-risk groups.

Clinical characteristics	TCGA cohort		GSE21257 cohort	
	Low risk	High risk	Low risk	High risk
No. of patients	44	42	27	26
Age (median)	14	14	16	16
Gender (%)				
Female	20 (45.5)	17 (40.5)	8 (29.6)	11 (42.3)
Male	24 (54.5)	25 (59.5)	19 (70.4)	15 (57.7)
Survival status				
OS days (median)	1729	719	2,342	974
Censored (%)	5 (11.2)	24 (57.1)	8 (29.6)	15 (57.7)

TCGA, The Cancer Genome Atlas; OS, overall survival.

### Construction and Validation of a Prognostic Nomogram

A prognostic nomogram, comprising clinical features such as gender and age, and the relative risk score based on the risk signature were constructed using the data set from the entire cohort. Calibration curves were plotted in both the entire cohort and GSE21257 cohort to evaluate the accuracy of the nomogram in predicting 1-, 2-, and 3-year overall survival.

### Functional Enrichment Analysis

The differentially expressed genes (DEGs) between the high- and low-risk groups in the gene expression matrix of the TCGA entire cohort were identified using the *limma* package in R. The genes with a threshold of | log2 (fold change) | > 0.5 and false discovery rate (FDR) <0.05 were regarded as DEGs and were subjected to Gene Ontology (GO) and Kyoto Encyclopedia of Genes and Genomes (KEGG) enrichment analyses using the *clusterProfiler* and *org.Hs.eg.db* packages in R.

### Gene Set Enrichment Analysis

The gene set enrichment analysis (GSEA) was performed between the high- and low-risk groups in the gene expression matrixes from the TCGA osteosarcoma cohort and the GSE21257 cohort using the GSEA 4.0.2 software. The enriched pathways were filtered with a threshold of nominal *p*-value <0.05 and normalized enrichment score (NES) >1.0.

### Single-Sample Gene Set Enrichment Analysis

The single-sample gene set enrichment analysis (ssGSEA) was performed using the *GSVA* package in R to quantify the level of immune cell infiltration and immune function. The patients in the entire cohort and GSE21257 cohort were imported to ssGSEA, and the scores of the immune cell and immune function in each sample were calculated and were further compared between the high- and low-risk groups.

### Statistical Analysis

All the statistical analyses were performed using R software version 4.1.0. The differences were considered to be significant at a *p*-value <0.05 if not specified.

## Results

### Identification of Prognosis-Related Zinc Finger Protein Genes in Osteosarcoma

**FIGURE 1:**
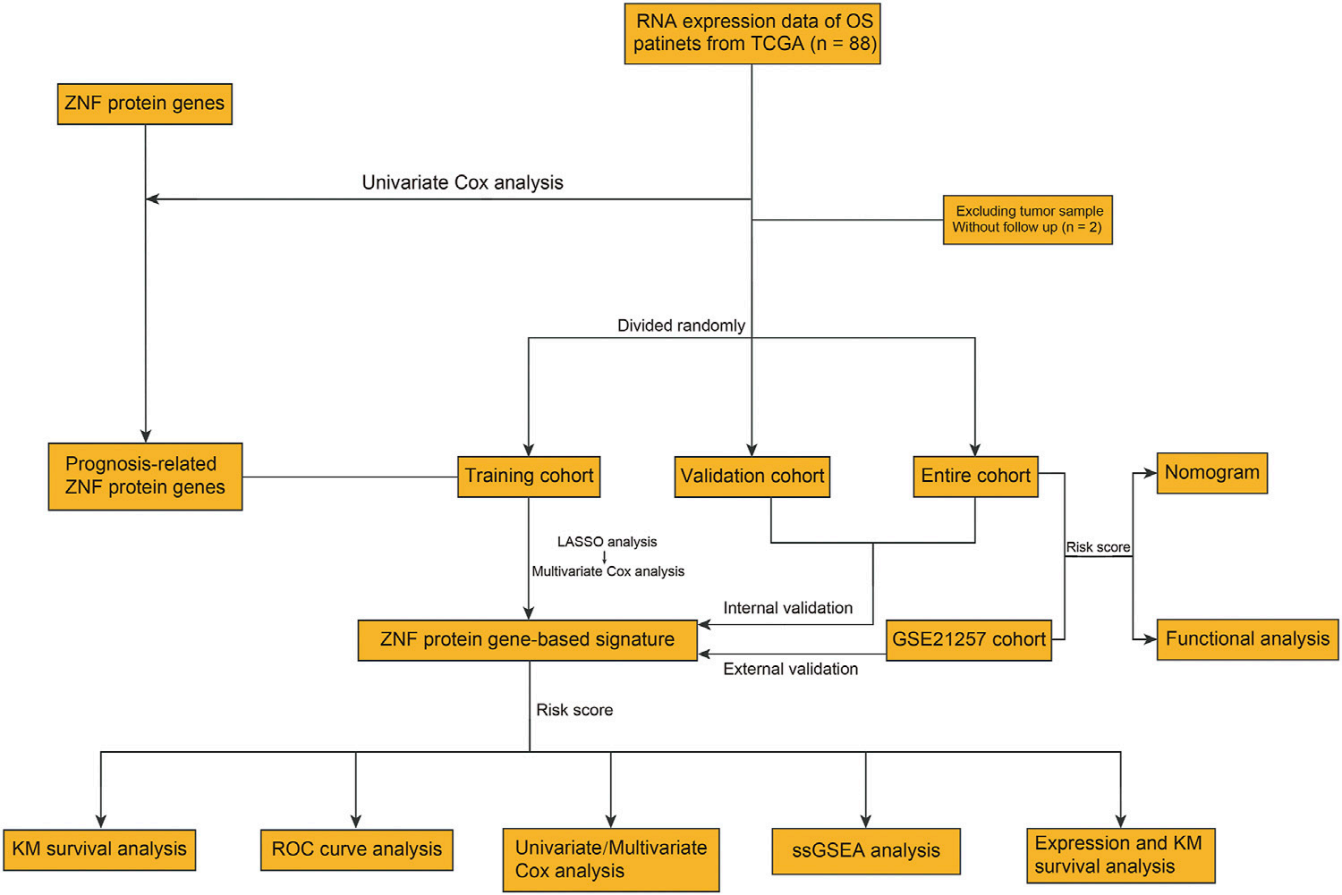
Workflow diagraph of data collection and analysis.

**FIGURE 2:**
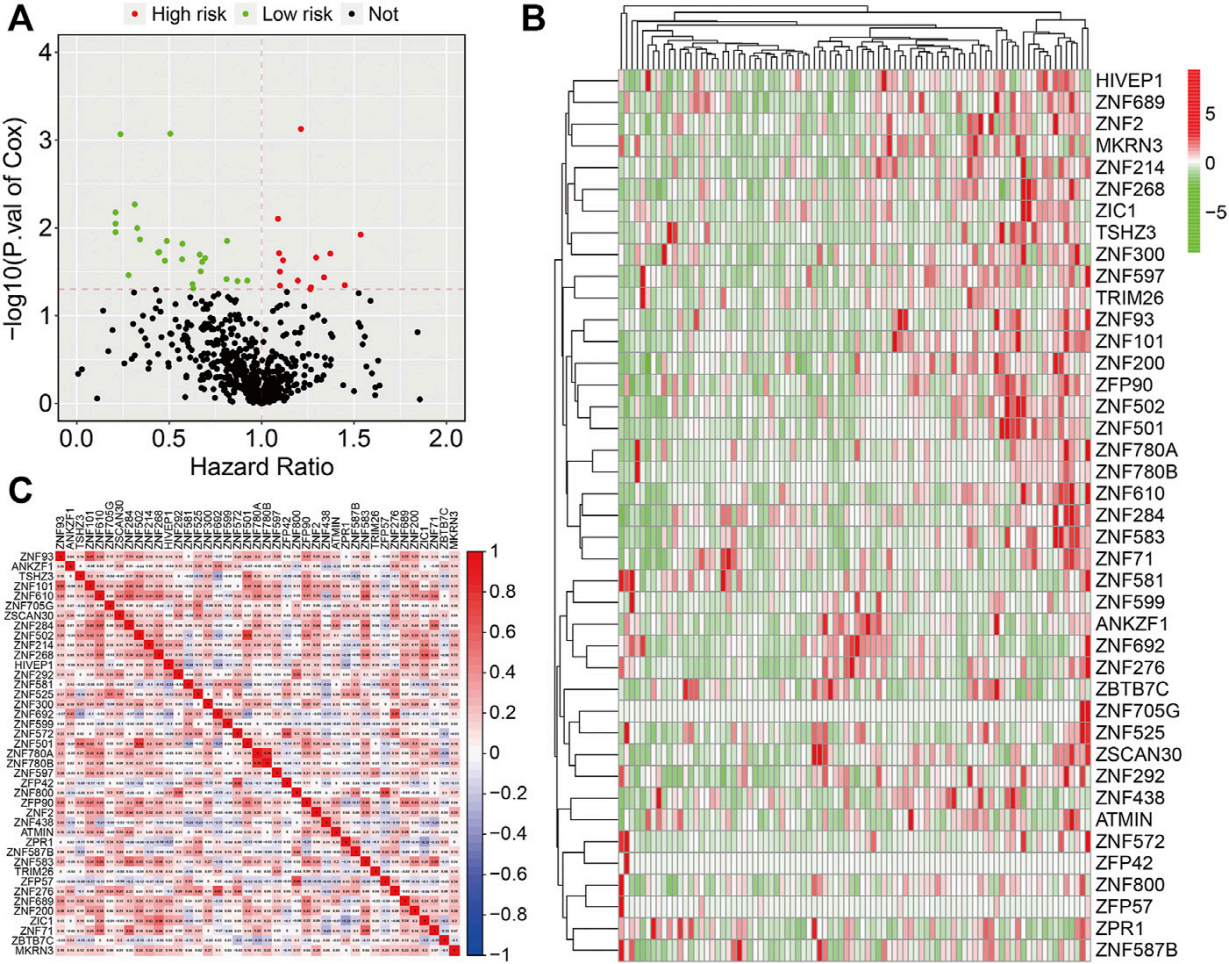
Identification of prognosis-related ZNF protein genes in osteosarcoma. **(A)** Prognosis-related ZNF protein genes were identified by performing univariate Cox regression analysis. **(B)** The expression profiles of the prognosis-related ZNF protein genes. **(C)** The correlation of the prognosis-related ZNF protein genes based on gene expression.

The flowchart of the present study is exhibited in Figure 1. First, we performed univariate Cox regression analysis to identify prognosis-related ZNF protein genes in osteosarcoma. As shown in Figure 2A, a total of 41 ZNF protein genes were significantly associated with the prognosis of osteosarcoma patients in the univariate Cox regression analysis, and these genes were identified as prognosis-related ZNF protein genes in osteosarcoma. Of these 41 ZNF protein genes, 16 were risk factors (hazard ratio (HR) > 1), while 25 were protective factors (HR < 1) in osteosarcoma. The expression profile of these 41 prognosis-related ZNF protein genes in osteosarcoma and the correlation of them are exhibited in Figures 2B,C.

### Construction of a Zinc Finger Protein Gene–Based Signature in Training Cohort

**FIGURE 3:**
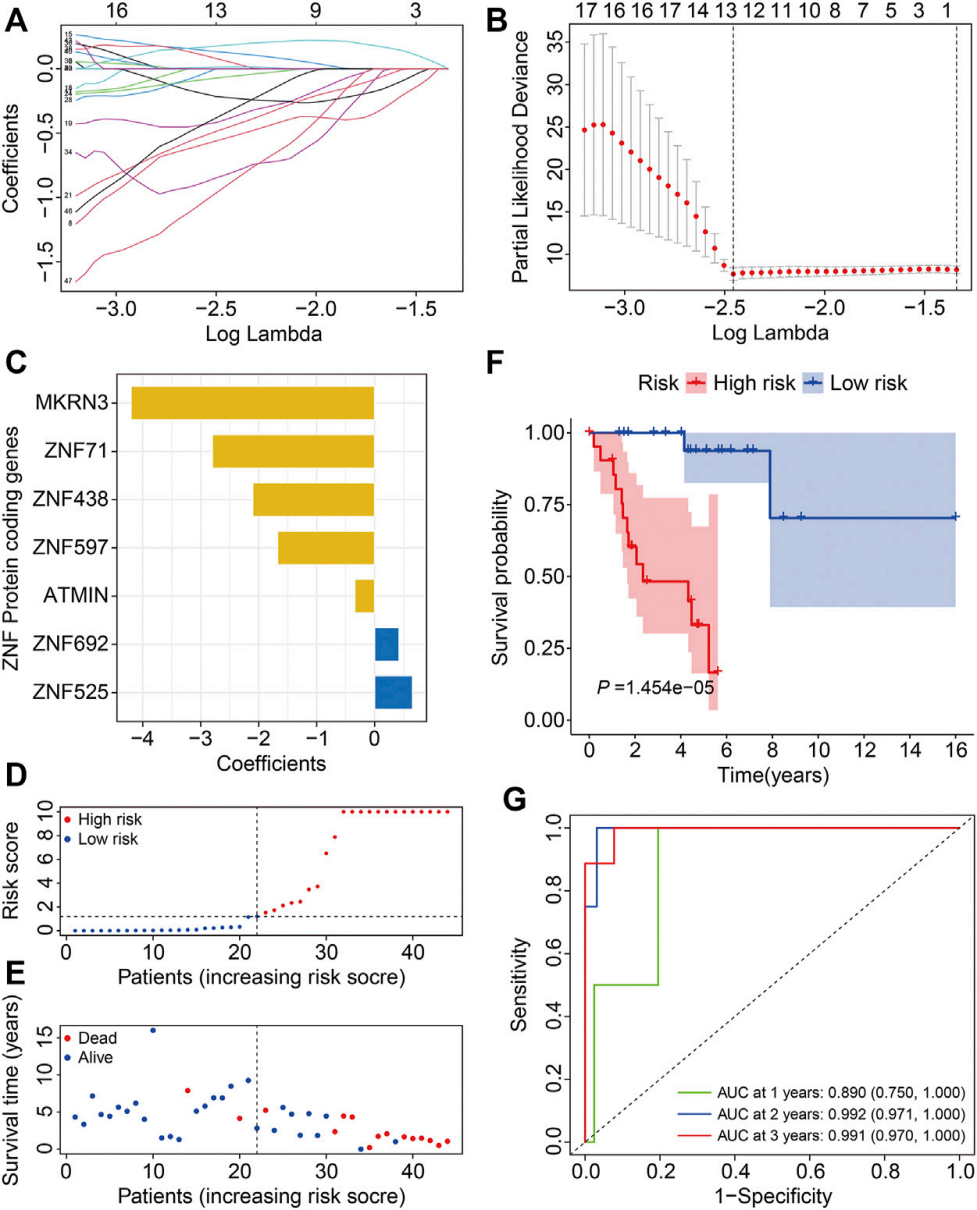
Construction of a ZNF protein gene–based signature in osteosarcoma training cohort. **(A, B)** Lasso regression and multivariable Cox regression analyses were performed to identify genes for the prognostic signature. **(C)** The coefficients of the seven genes. **(D)** Risk score distribution. **(E)** Survival time and survival status of patients in high- and low-risk groups. **(F)** Kaplan–Meier survival analysis in high- and low-risk groups. **(G)** Time-dependent ROC curves for 1-, 2-, and 3-year survival.

In the training cohort, we performed LASSO regression analysis to eliminate overfitting and then conducted multivariate Cox regression analysis to calculate the regression coefficients of the selected genes (Figures 3A,B). Finally, we developed a novel prognostic signature based on seven ZNF protein genes in osteosarcoma. The seven genes included *MKRN3*, *ZNF71*, *ZNF438*, *ZNF597*, *ATMIN*, *ZNF692*, and *ZNF525*, and the coefficients of these are shown in Figure 3C. The risk scores of the patients in the training cohort were calculated using the following formula: risk score = *MKRN* × (−4.202) + *ZNF71* × (−2.800) + *ZNF438* × (−2.102) + *ZNF597* × (−1.675) + *ATMIN* × (−0.343) + *ZNF692* × 0.420 + *ZNF525* × (0.652), and it allowed patients to be classified into high- and low-risk groups based on the median value of the risk score. The risk score distribution of patients in the high- and low-risk groups is exhibited in Figure 3D. The survival time of the patients in the high-risk group tended to be worse than that in the low-risk group (Figure 3E). Moreover, the Kaplan–Meier survival analysis revealed that the survival probability of the patients in the high-risk group was dramatically lower than that in the low-risk group (Figure 3F). The time-dependent ROC curves were plotted, and the areas under the curve (AUC) for 1-, 2-, and 3-year survival were 0.890, 0.992, and 0.991, respectively (Figure 3G).

### Validation of the Zinc Finger Protein Gene–Based Signature in Internal and External Cohorts

**FIGURE 4:**
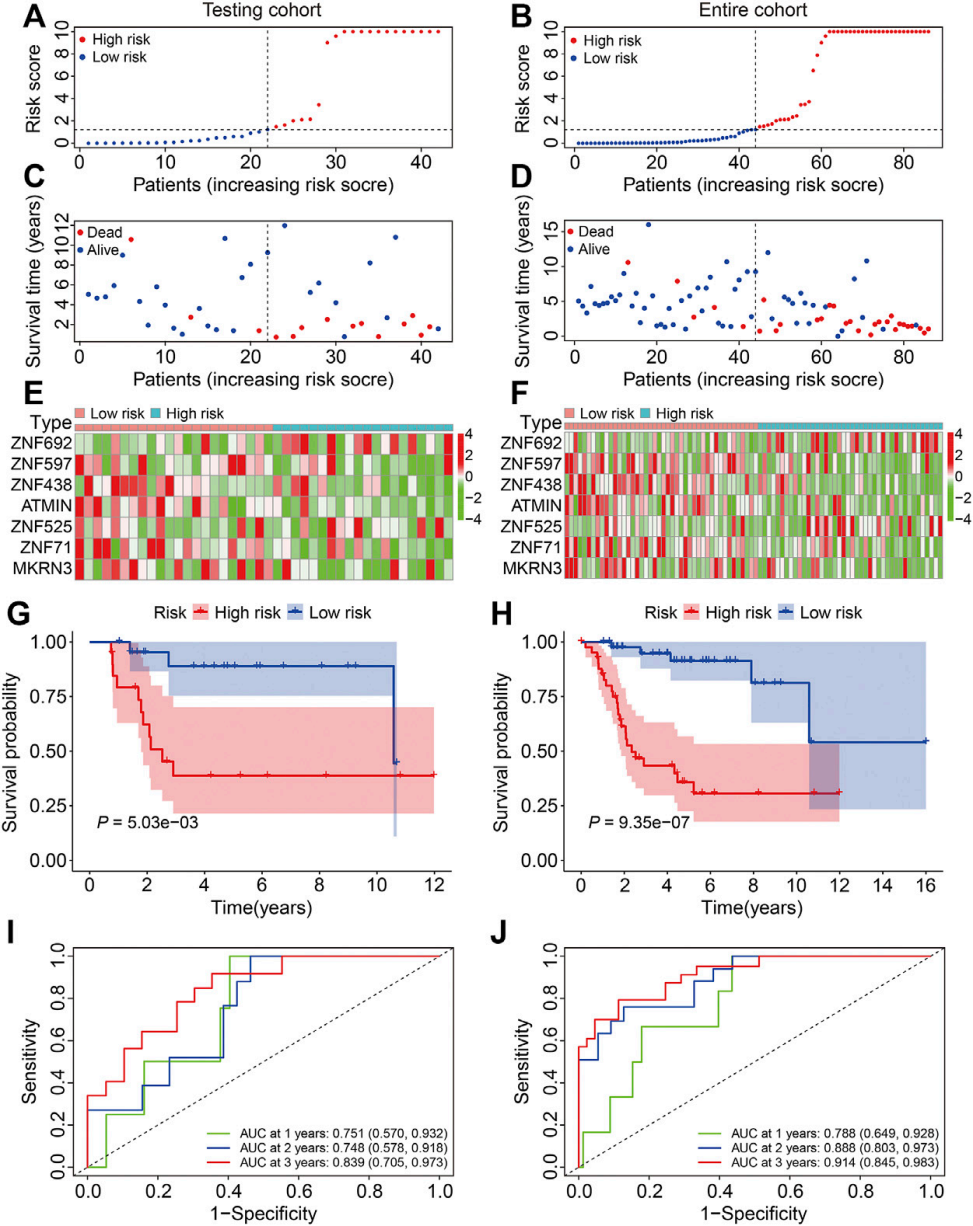
Validation of the ZNF protein gene-based signature in osteosarcoma testing and entire cohorts. **(A, B)** Risk score distribution of patients in testing and entire cohorts. **(C, D)** Survival time and survival status of patients in testing and entire cohorts. **(E, F)** The expression profile of the seven ZNF protein genes in testing and entire cohorts. **(G, H)** Kaplan–Meier survival analysis of high- and low-risk patients in testing and entire cohorts. **(I, J)** Time-dependent ROC curves for 1-, 2-, and 3-year survival in testing and entire cohorts.

**FIGURE 5:**
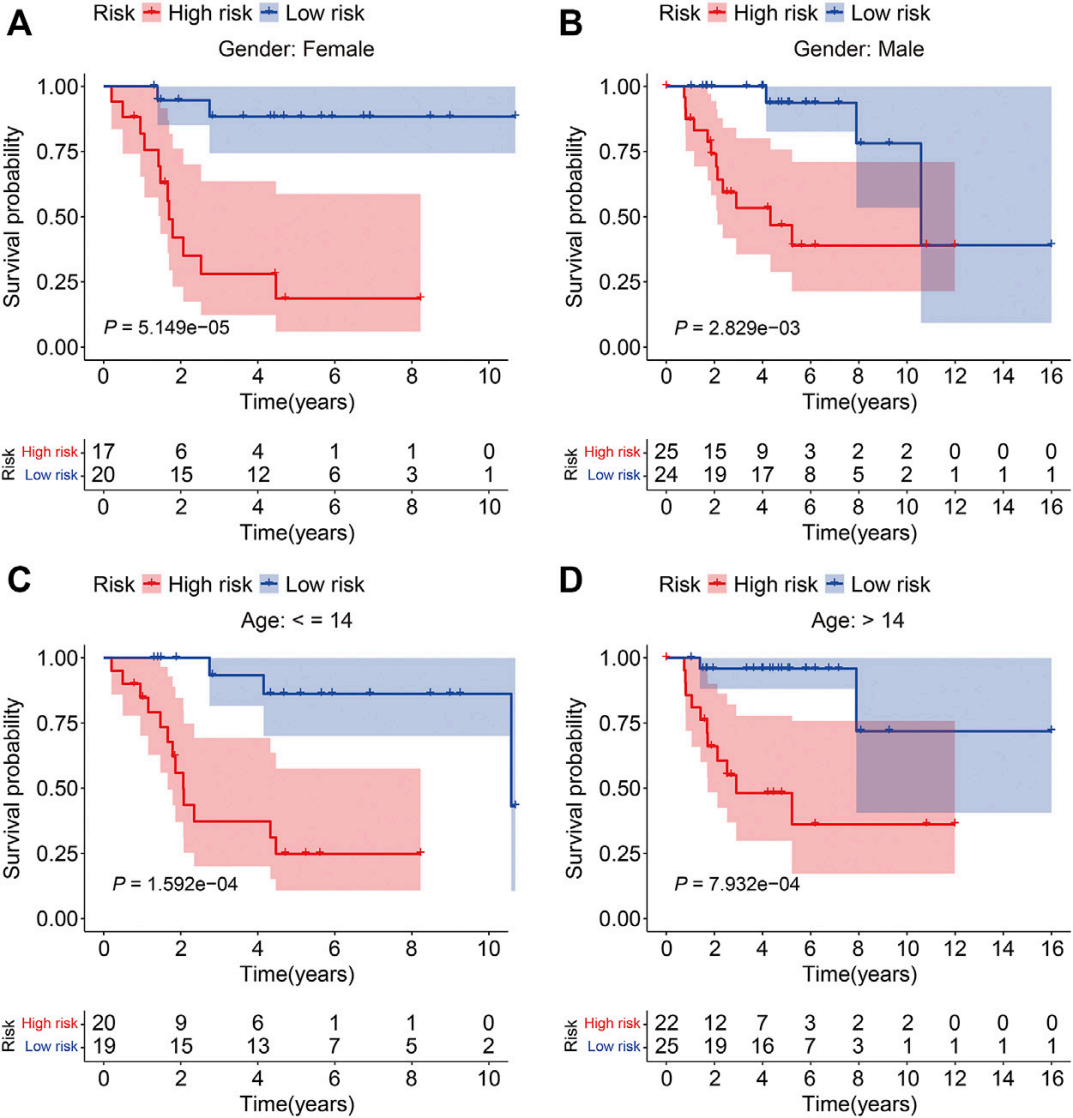
Kaplan–Meier survival analysis in subgroups stratified by gender **(A, B)** or age **(C, D)** in the TCGA cohort.

To verify the credibility of our ZNF protein gene–based signature in predicting the prognosis of patients with osteosarcoma, the internal cohorts, including the testing cohort and entire cohort, were employed. Using the same aforementioned formula, the risk scores were calculated, and it allowed patients to be stratified into the high- and low-risk groups with the median risk score in the training cohort as the cutoff value. The risk score distribution of the high- and low-risk patients in the testing and entire cohorts is exhibited in Figures 4A,B. The survival time and status of the patients in the testing and entire cohort are shown in Figures 4C,D, and it seems that the mortality rate of the patients in the high-risk group was higher than in the low-risk group. The expression profiles of the seven ZNF protein genes are displayed in Figures 4E,F. The Kaplan–Meier survival curves showed that the prognosis of the patients in the high-risk group was worse than in the low-risk group in both the testing and entire cohorts (Figures 4G,H). The AUC values for 1-, 2-, and 3-year survival in the testing cohort were 0.751, 0.748, and 0.839, respectively (Figure 4I), and were 0.788, 0.888, and 0.914, respectively, in the entire cohort (Figure 4J). To further evaluate the predictive power of the ZNF protein gene–based prognostic signature, the Kaplan–Meier survival analyses were performed in various subgroups stratified by gender and age, and the results suggested that in all these subgroups, the overall survival of the patients in the high-risk group was worse than in the low-risk group (Figures 5A–D).

**FIGURE 6:**
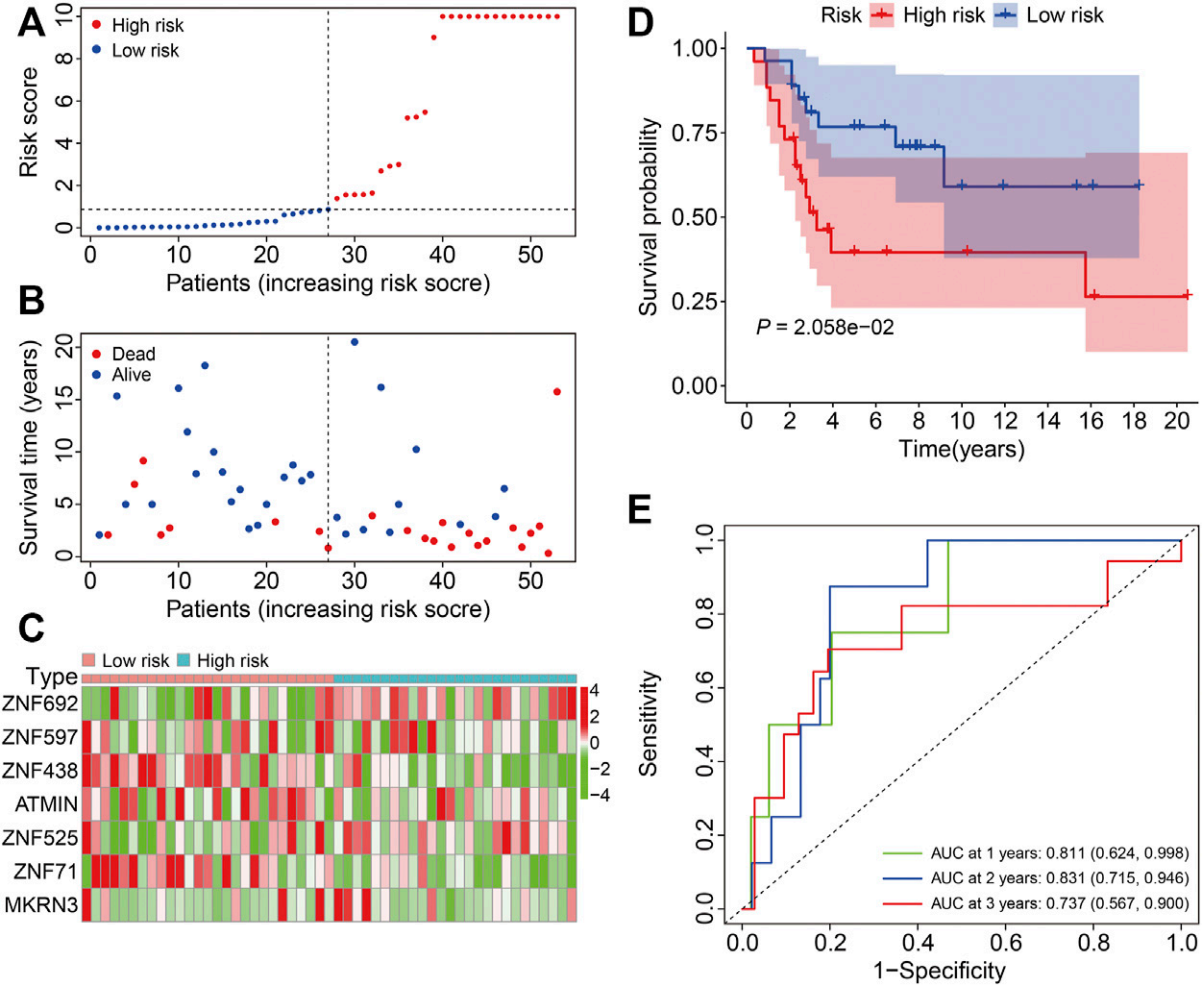
Validation of the ZNF protein gene-based signature in GSE21257 cohort. **(A)** Risk score distribution of patient in the GSE21257 cohort. **(B)** Survival time and survival status of patients in the GSE21257 cohort. **(C)** The expression profile of the seven ZNF protein genes in GSE21257 cohort. **(D)** Kaplan–Meier survival analysis of high- and low-risk patients in the GSE21257 cohort. **(E)** Time-dependent ROC curves for 1-, 2-, and 3-year survival in the GSE21257 cohort.

To assess the general applicability of the ZNF protein gene–based prognostic signature, the external GSE21257 cohort was enrolled in the subsequent analysis. The risk scores of the patients in the GSE21257 cohort were calculated, and the patients were then divided into the high- and low-risk groups according to the median risk score in the training cohort (Figure 6A). The survival time and survival status of the patients in the GSE21257 cohort are exhibited in Figure 6B, and the result indicated that the patients of the high-risk group harbored a higher mortality rate than those in the low-risk group. The expression profiles of the seven ZNF protein genes in the GSE21257 gene expression matrixes are shown in Figure 6C. The Kaplan–Meier survival analysis suggested that the overall survival of the patients in the high-risk group continued to be worse than in the low-risk group (Figure 6D). The AUC values for 1-, 2-, and 3-year survival were 0.811, 0.831, and 0.737, respectively (Figure 6E). Taken together, these internal and external cohorts confirmed the satisfactory accuracy and generalizability of the ZNF protein gene–based prognostic signature.

### The Prognostic Independence of the Zinc Finger Protein Gene–Based Signature and Construction of a Nomogram in Osteosarcoma

**FIGURE 7:**
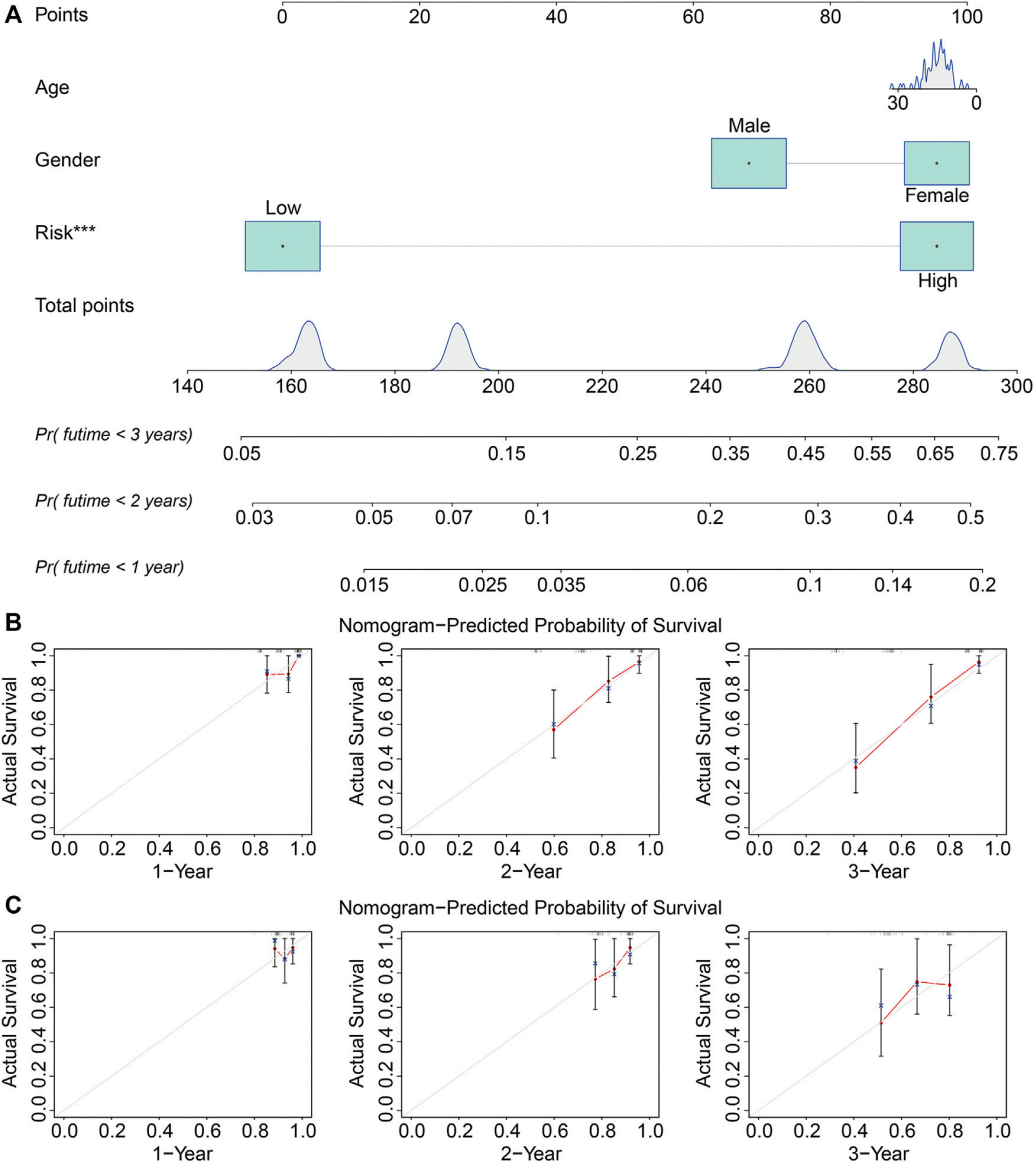
Construction and validation of a nomogram in TCGA and GSE21257 cohorts. **(A)** Nomogram of clinical feature including age and gender, and relative risk based on the signature. **(B, C)** Calibration plots of the nomogram for predicting the probability of overall survival at 1, 2, and 3 years in TCGA and GSE21257 cohorts.

The univariate and multivariate Cox regression analyses were utilized to evaluate the independent predictive power of the ZNF protein gene–based signature. As shown in Table 2, the results indicated that the risk score based on the seven-gene signature was the only independent prognostic biomarker in osteosarcoma. Moreover, we constructed a prognostic nomogram using the gene expression matrixes of the entire cohort and corresponding clinical information. The total point score was calculated based on the relative risk score and clinical features including gender and age. The probability of the 1-, 2-, and 3-year overall survival was reflected by the total points and is shown in Figure 7A. To evaluate the validity of the nomogram, the calibration plots were visualized in both TCGA entire cohort and the GSE21257 cohort. The results showed that the predicted overall survival at 1, 2, and 3 years was consistent with the actual overall survival at 1, 2, and 3 years (Figures 7B,C), suggesting that the nomogram had great accuracy in predicting the overall survival in osteosarcoma.

**TABLE 2:** Univariable and multivariable analysis of the ZNF protein gene–based signature and clinical factors in the TCGA osteosarcoma cohort.

Variables	Univariable analysis				Multivariable analysis			
	HR	95% CI of HR		P	HR	95% CI of HR		P
		Lower	Upper			Lower	Upper	
Gender (female vs male)	0.6811	0.3276	1.4159	0.3037	1.0225	0.4616	2.2650	0.9563
Age (≤14 vs >14)	0.6515	0.3132	1.3553	0.2516	0.6041	0.2769	1.3179	0.2054
Risk score	1.0035	1.0022	1.0047	0.0000	1.0036	1.0022	1.0049	0.0000

HR, hazard ratio; CI, confidence interval.

### Identification of Risk-Related Differentially Expressed Genes and Functional Enrichment Analysis

**FIGURE 8:**
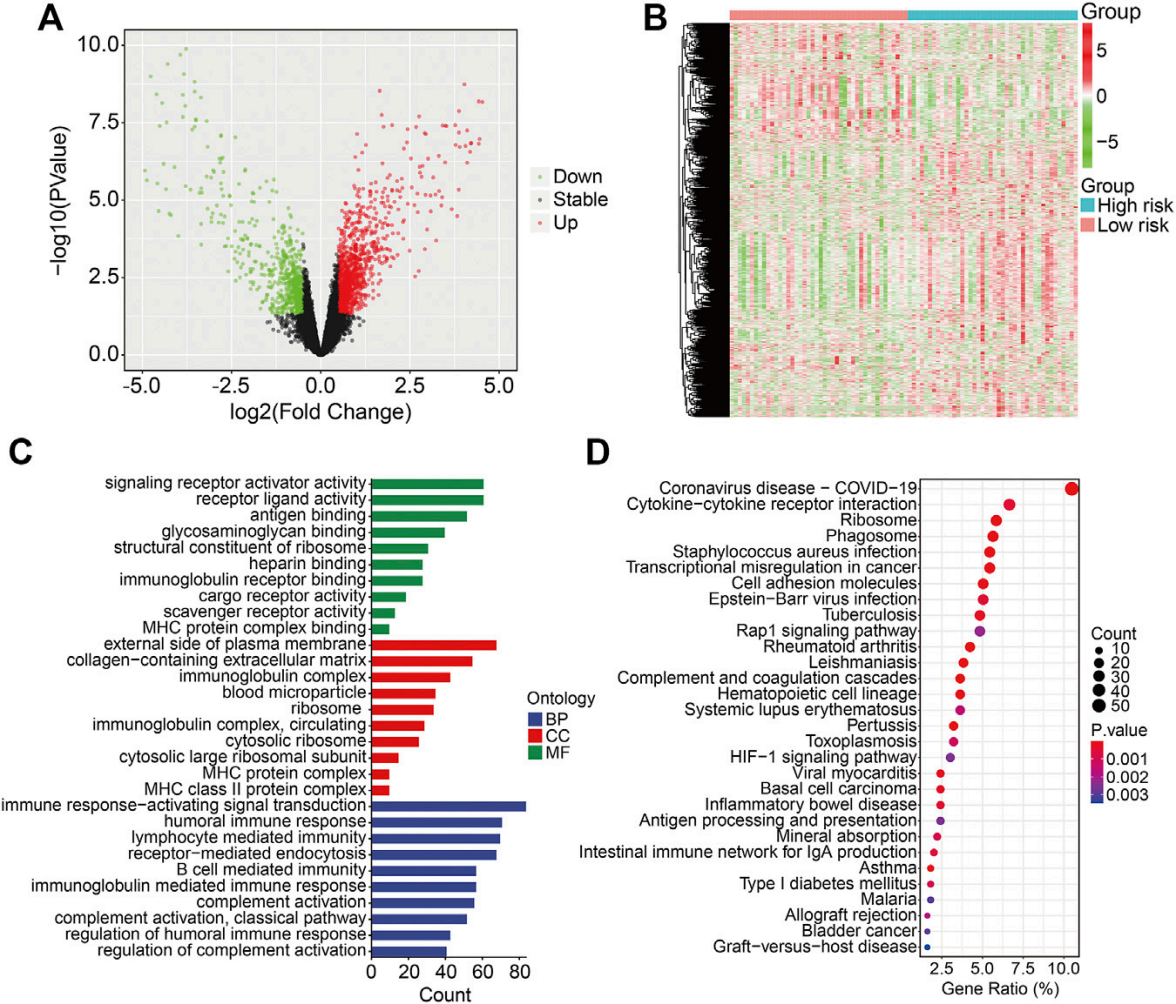
Identification of risk-related differentially expressed genes and functional enrichment analysis. **(A)** Vocal plot exhibited risk-related differentially expressed genes. **(B)** Expression profiles of genes differentially expressed between high- and low-risk groups in gene matrixes of the TCGA cohort. **(C, D)** GO and KEGG enrichment analysis of risk-related differentially expressed genes.

To reveal the underlying mechanism related to the ZNF protein gene–based signature, we identified genes that were differentially expressed between the high- and low-risk groups using the *limma* package in R. A total of 1,819 DEGs were screened out with the criteria of | log2 (fold change) | > 0.5 and an FDR < 0.05 (Figure 8A). Of the 1,819 DEGs, 1,178 genes were upregulated and 641 genes were downregulated in the high-risk group. The expression profiles of these 1,819 genes in the high- and low-risk groups are exhibited in Figure 8B. Then, we performed GO and KEGG enrichment analyses on the 1,819 DEGs. In biological processes, DEGs mainly participate in the MHC class II protein complex, immune response–activating signal transduction, humoral immune response, lymphocyte-mediated immunity, and receptor-mediated endocytosis. In terms of molecular functions, DEGs are primarily enriched in signaling receptor activator activity, receptor ligand activity, antigen binding, glycosaminoglycan binding, and structural constituent of the ribosome. In cellular components, enriched terms were mainly related to the external side of the plasma membrane, collagen-containing extracellular matrix, and immunoglobulin complex (Figure 8C). The KEGG enrichment analysis revealed that the DEGs were primarily enriched in cytokine–cytokine receptor interaction, the ribosomes, the phagosomes, transcriptional mis-regulation in cancer, and cell adhesion molecules (Figure 8D).

### Gene Set Enrichment Analysis and Immune Score Analysis

**FIGURE 9:**
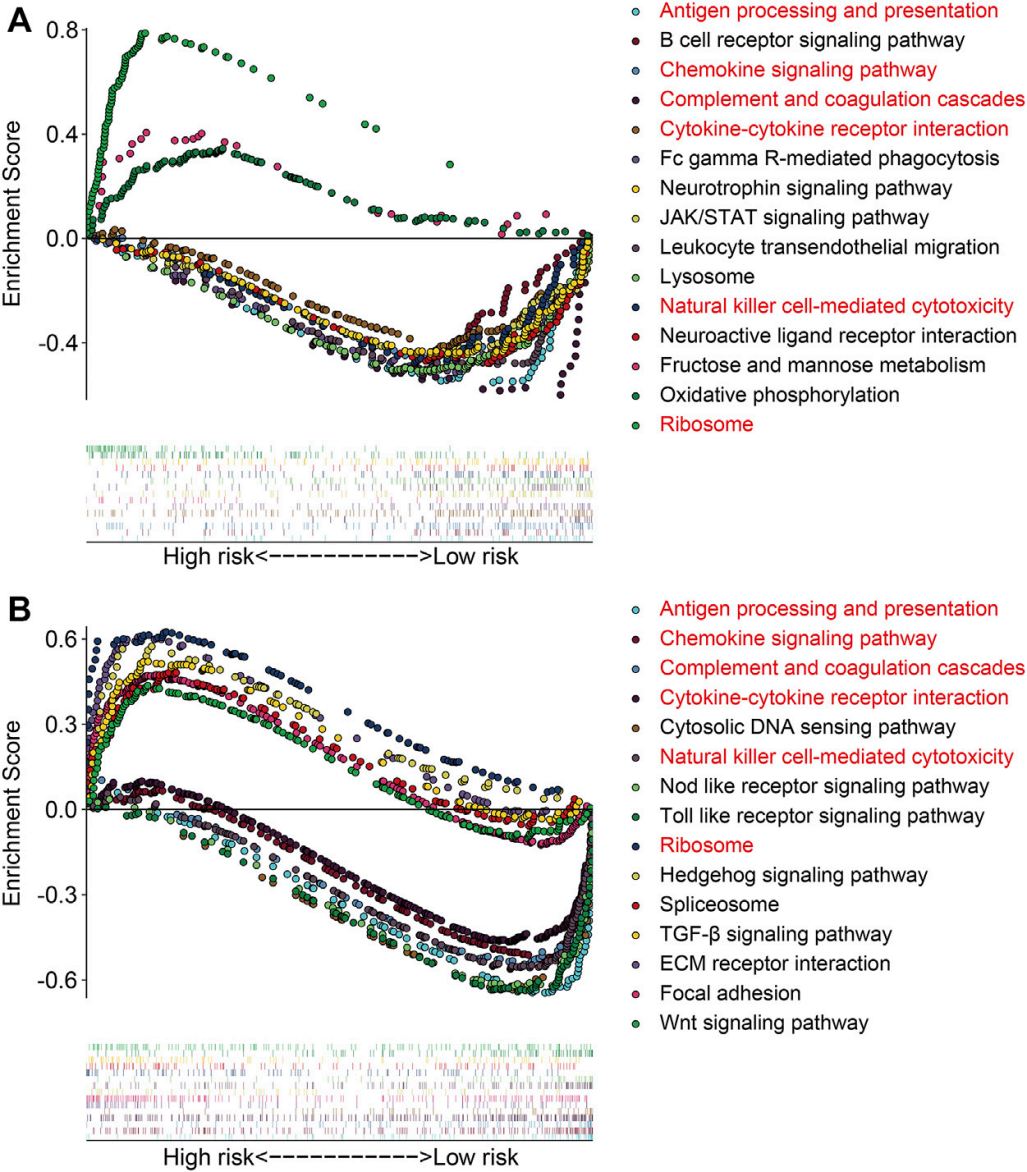
Gene set enrichment analysis between high- and low-risk groups in gene expression matrixes of the TCGA cohort **(A)** and GSE21257 cohort **(B)**.

**FIGURE 10:**
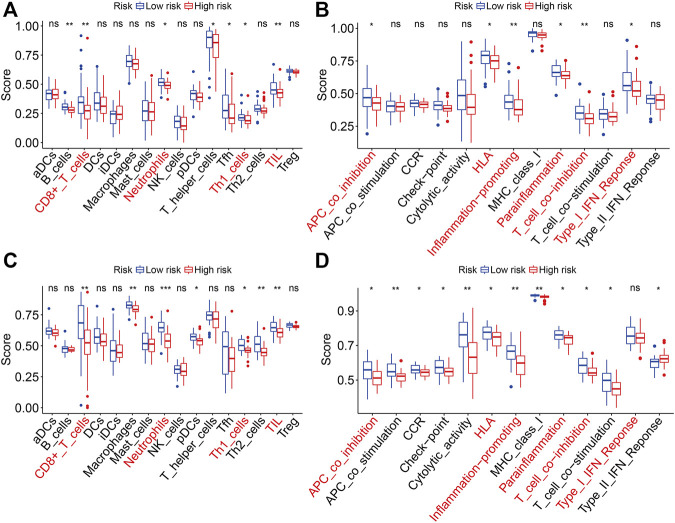
Comparison of ssGSEA scores between the high- and low-risk groups in TCGA and GSE21257 cohorts. **(A, B)** Scores of 16 immune cell types in TCGA and GSE21257 cohorts. **(C, D)** Scores of related immune functions in TCGA and GSE21257 cohorts.

The gene set enrichment analysis (GSEA) was performed between the high- and low-risk groups in TCGA and GSE21257 gene expression matrixes with a threshold of NES >1 and FDR <0.05. As shown in Figures 9A,B, the immune-related pathways including antigen processing and presentation, chemokine signaling pathway, complement and coagulation cascades, cytokine–cytokine receptor interaction, natural killer cell–mediated cytotoxicity, and ribosomes were significantly enriched in the two gene expression matrixes. These analyses connected the ZNF protein gene–based signature with the immune-related pathways. We further compared the enrichment scores of various immune cell subpopulations and related immune functions in the high- and low-risk groups by performing ssGSEA. As shown in Figures 10A,B, the scores of diverse immune cell subpopulations including the CD8^+^ T cells, neutrophils, Th1 cells, and TILs were significantly lower in the high-risk group than in the low-risk group in the two gene expression matrixes. As for the related immune functions, there were significant differences between the high- and low-risk groups for APC co-inhibition, HLA, inflammation promoting, para-inflammation, T-cell co-inhibition, and type I IFN response (Figures 10C,D).

### Expression and Kaplan–Meier Survival Analysis of the Seven Zinc Finger Protein Genes

**FIGURE 11:**
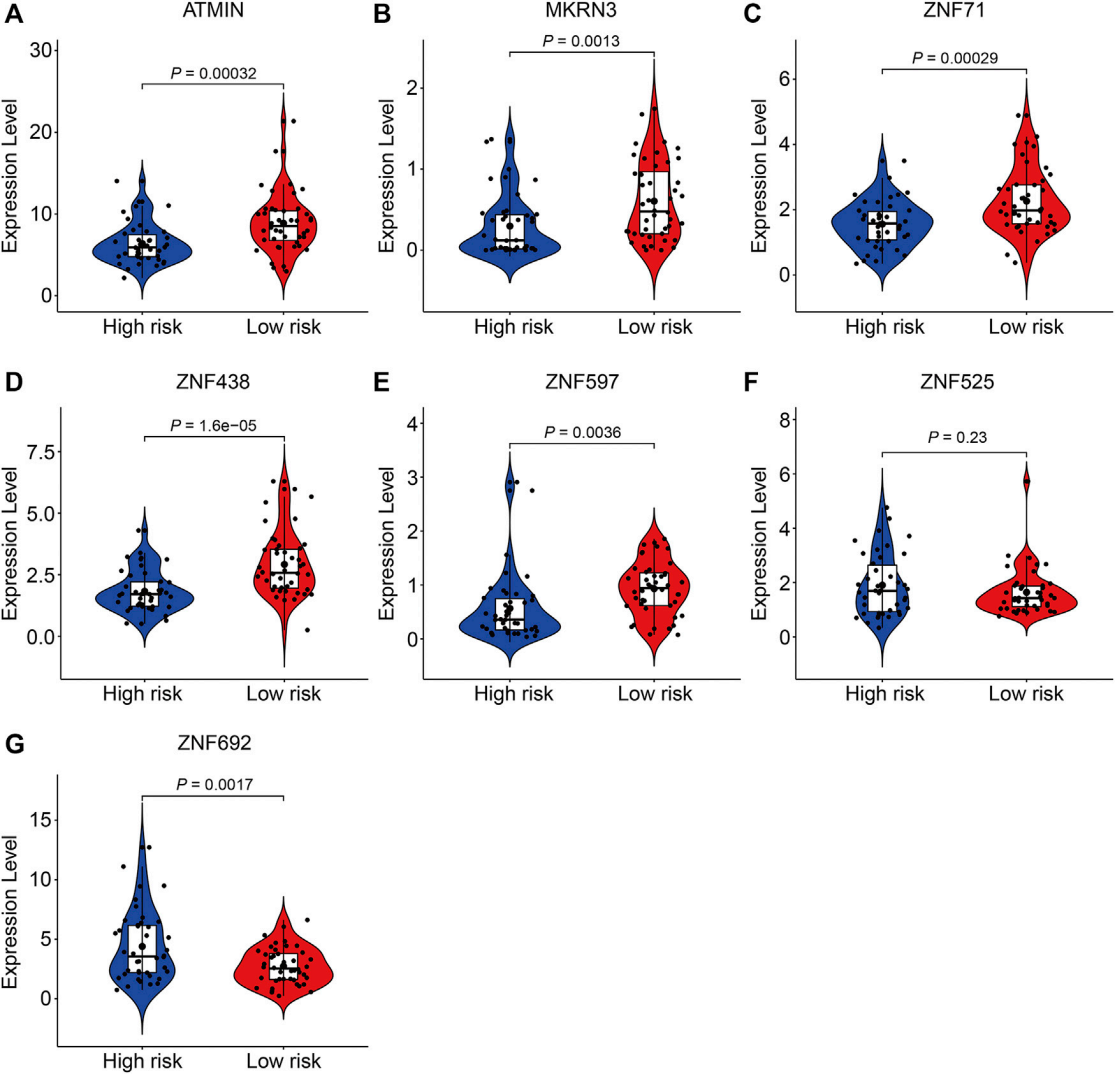
Comparison of the expression of *ATMIN* **(A)**, *MKRN3* **(B)**, *ZNF71* **(C)**, *ZNF438* **(D)**, *ZNF597* **(E)**, *ZNF525* **(F)**, and *ZNF692* **(G)** in high- and low-risk groups.

**FIGURE 12:**
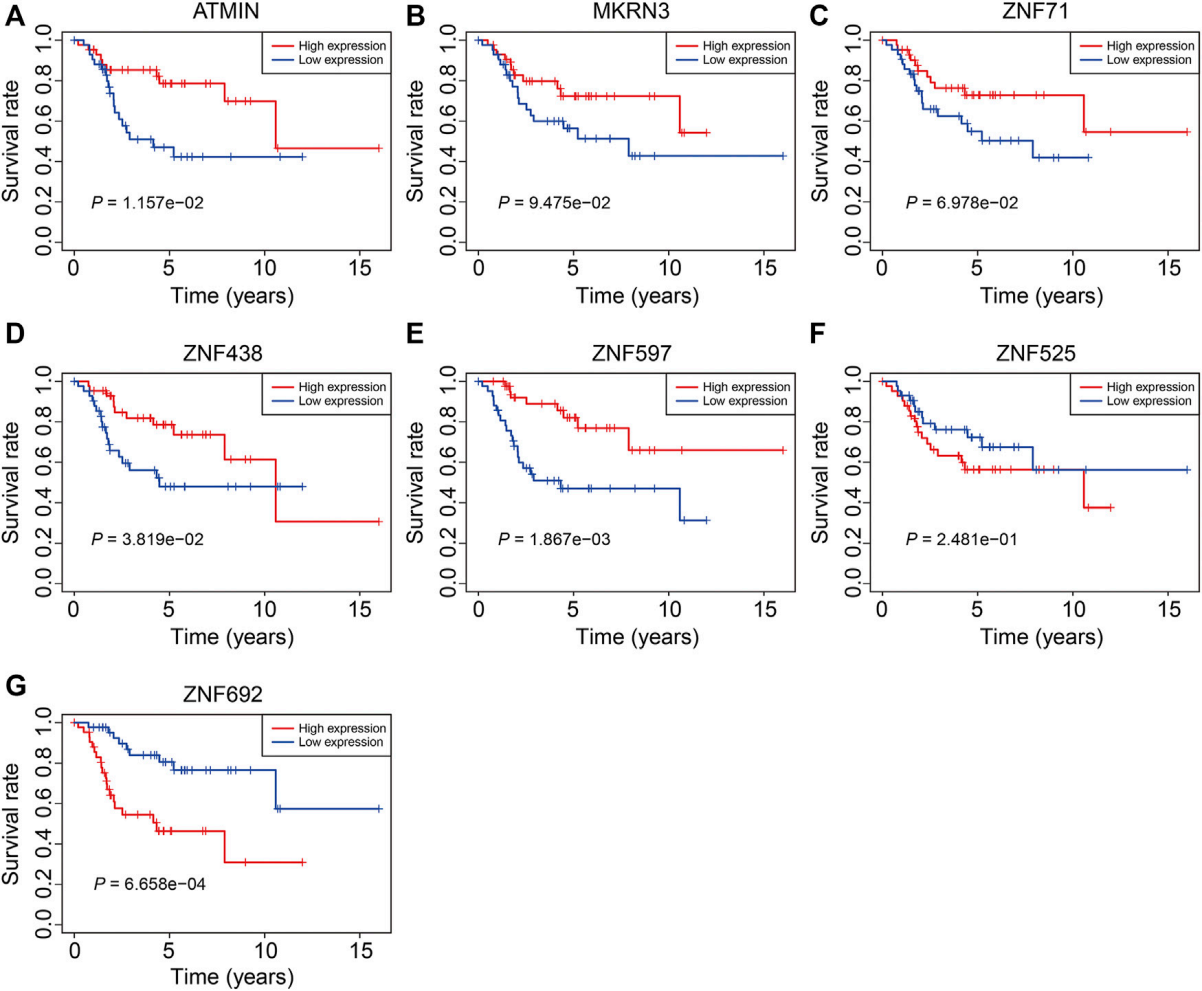
Kaplan–Meier survival analysis of *ATMIN* **(A)**, *MKRN3* **(B)**, *ZNF71* **(C)**, *ZNF438* **(D)**, *ZNF597* **(E)**, *ZNF525* **(F)**, and *ZNF692* **(G)** in the TCGA cohort.

Finally, we performed expression and Kaplan–Meier survival analyses of the seven ZNF protein genes using the data set from the TCGA entire cohort. As shown in Figures 11A–E, the expressions of *ATMIN*, *MKRN3*, *ZNF71*, *ZNF438*, and *ZNF597* were lower in the high-risk group than in the low-risk group. Conversely, the expressions of *ZNF525* and *ZNF692* were higher in the high-risk group than in the low risk-group (Figures 11F,G). Moreover, the Kaplan–Meier survival analysis suggested that the lower expressions of *ATMIN*, *ZNF438*, and *ZNF597* and higher expression of *ZNF692* were associated with worse overall survival in osteosarcoma, while the expressions of *MKRN3*, *ZNF71*, and *ZNF525* were not significantly related to the prognosis of patients (Figures 12A–G).

## Discussion

Osteosarcoma is a highly heterogeneous malignant tumor that mainly occurs in the metaphysis of the long bone (Saraf et al., 2018). The clinical outcome of patients with osteosarcoma varies even when patients are treated under the standard management (Rickel et al., 2017). Predicting the prognosis of patients in osteosarcoma accurately is of important clinical value, which may be helpful for risk stratification and clinical decision-making and for guiding individual treatment. Up to now, the most widely used prognostic markers for osteosarcoma include tumor features such as the AJCC-TNM classification and clinical factors, including gender, age, and site (Hattinger et al., 2018). However, the sensitivity and accuracy of these factors in predicting the prognosis of patients with osteosarcoma are limited. Recently, reanalyzing data sets from public databases using bioinformatic methods provides an avenue for the identification of prognostic biomarkers in malignant tumors. For example, Zhang et al. (2021) constructed an autophagy-related long noncoding RNA signature in osteosarcoma using data sets from the TCGA database and confirmed the accuracy of the signature in predicting the clinical outcomes of patients with osteosarcoma.

Given the critical role of ZNF proteins in oncogenesis and progression, here we conducted a comprehensive analysis of the ZNF protein genes in osteosarcoma and identified prognosis-related ZNF protein genes. Furthermore, these genes were utilized to constructed a seven-gene prognostic signature that allowed patients to be stratified into high- and low-risk groups. The Kaplan–Meier survival analyses in the internal and external cohorts confirmed that the patients of the high-risk group had worse overall survival than these of the low-risk group, suggesting the satisfactory accuracy and generalizability of the ZNF protein gene–based signature in predicting the prognosis of the osteosarcoma patients. Moreover, time-dependent ROC curve analysis confirmed the sensitivity and specificity of the ZNF protein gene–based signature. Compared with the previously reported prognostic signatures in osteosarcoma (Zhang et al., 2021), we found that the specificity and accuracy of our ZNF protein gene–based signature were superior to these prognostic signatures, since the AUC values were higher in our research. Moreover, a prognostic nomogram comprising clinical features such as gender and age and the relative risk score based on the risk signature was constructed, and it might be helpful for clinical decision-making and designing personalized management. To identify the underlying mechanism associated with the ZNF protein gene–based signature, genes differentially expressed between the high- and low-risk groups were screened out and subjected to functional enrichment analyses. The results suggested that the DEGs participated in immune-related biological processes such as MHC class II protein complex, immune response–activating signal transduction, humoral immune response, and lymphocyte-mediated immunity. Interestingly, the gene set enrichment analysis also connected immunity with the ZNF protein gene–based signature. We also found that the risk-related DEGs were enriched in COVID-19–related pathways. Subsequently, we further compared enrichment scores of various immune cell subpopulations and related immune functions between the high- and low-risk groups. We found that the scores of diverse immune cell subpopulations, including CD8^+^ T cells, neutrophils, Th1 cells, and TILs, were significantly lower in the high-risk group than in the low-risk group. As for the related immune functions, there were significant differences between the high- and low-risk groups for APC co-inhibition, HLA, inflammation promoting, para-inflammation, T-cell co-inhibition, and type I IFN response. These analyses indicated the immune suppressive states of the high-risk group, and we might speculate that patients in the high-risk group may not respond to immune checkpoint inhibitors (Zhang et al., 2020). Therefore, our ZNF protein gene–based signature could be utilized to predict the outcome of patients treated with immune therapy.

Of the seven genes comprising our prognostic signature, the expressions of *ATMIN*, *ZNF438*, and *ZNF597* were lower in the high-risk group, and lower expressions of *ATMIN*, *ZNF438*, and *ZNF597* predicted worse overall survival in patients with osteosarcoma, suggesting that *ATMIN*, *ZNF438*, and *ZNF597* might act as protective factors in osteosarcoma. Up to now, few researches reported the role of *ZNF438* and *ZNF597* in tumors. *ATMIN*, also named as *ZNF822*, functions as a cofactor of ATM and is required for the activation of the ATM signaling pathway (Kanu et al., 2010; Liu et al., 2017). ATM participates in the maintenance of genomic stability and DNA damage repair (Prochazkova et al., 2015; Li et al., 2019) and has been implicated as a tumor suppressor in various cancer types including lung adenocarcinoma (Foster et al., 2019), B-cell lymphoma (Loizou et al., 2011), and colorectal cancer (Li et al., 2021). However, little is known about the role of *ATMIN* in osteosarcoma, and based on our analysis, we can speculate that *ATMIN* functions as a tumor suppressor in osteosarcoma. The effect of *ATMIN* on osteosarcoma cell proliferation, migration, and invasion and the underlying mechanism need to be further investigated. *MKRN3*, also known as *ZNF127*, acts as an E3 ligase and is responsible for the degradation of target proteins through ubiquitination processes (Abreu et al., 2015). *MKRN3* is frequently mutated in non–small-cell lung cancers (NSCLCs), and genomic aberrations of *MKRN3* are found to be enriched in NSCLC samples harboring *KRAS* mutations (Li et al., 2021). Lower expression of *MKRN3* is associated with worse overall survival in patients with NSCLCs. Reconstitution of *MKRN3* in NSCLC cells directly abrogates tumor growth *in vitro* and *in vivo*, suggesting *MKRN3* functions as a tumor suppressor in NSCLCs. Here, we found *MKRN3* expression was lower in the high-risk group, and the lower expression of *MKRN3* had a tendency to be associated with a worse overall survival in osteosarcoma. Therefore, *MKRN3* might be a protective factor in osteosarcoma. *ZNF692*, also known as *AREBP* and *Zfp692*, was reported to have an important role in cell proliferation, invasion, and metastasis of lung and colon adenocarcinomas (Zhang et al., 2017; Xing et al., 2019). *ZNF692* was upregulated in cervical cancer tissues, and its overexpression was associated with poor clinicopathological characteristics in patients with cervical cancer (Zhu et al., 2019). These reports indicate that *ZNF692* might be an oncogene in tumors, and consistently, we found that *ZNF692* was higher in the high-risk group than in the low risk-group, and the higher expression of *ZNF692* was associated with worse overall survival in osteosarcoma. Therefore, it is reasonable to speculate that *ZNF692* might have an effect on the malignant behaviors of osteosarcoma cells.

There are some limitations in our study. First, the ZNF protein gene–based signature was only validated using cohorts from the public database, the accuracy and generalizability of the prognostic signature should also be verified in using prospective data, and we are collecting osteosarcoma patients to get our own cohort. Second, the role of the seven ZNF protein genes in osteosarcoma cells should be further explored. We will design specific siRNAs and construct overexpression plasmid for these ZNF protein genes and perform functional experiments to explore the effect of these genes on cell proliferation, migration, invasion, cell cycle, cell apoptosis, and stemness.

In all, we here comprehensively analyzed ZNF protein genes in osteosarcoma and constructed a prognostic signature based on seven ZNF protein genes. The signature is a novel and clinically useful prognostic biomarker for osteosarcoma patients.

## Data Availability

The original contributions presented in the study are included in the article/supplementary material, and further inquiries can be directed to the corresponding author.
